# Heat shock interferes with the amino acid metabolism of bovine cumulus-oocyte complexes in vitro: a multistep analysis

**DOI:** 10.1007/s00726-023-03370-6

**Published:** 2024-01-27

**Authors:** Hayder Radhi Hussein Mzedawee, Rasoul Kowsar, Reza Moradi-Hajidavaloo, Roya Shiasi-Sardoabi, Khaled Sadeghi, Mohammad Hossein Nasr-Esfahani, Mehdi Hajian

**Affiliations:** 1 Department of Animal Sciences, College of Agriculture, Isfahan University of Technology, Isfahan, Iran; 2 Department of Animal Biotechnology, Reproductive Biomedicine Research Center, Royan Institute for Biotechnology, ACECR, Isfahan, Iran

**Keywords:** Heat shock, Bovine cumulus-oocyte complexes, Amino acids, In vitro maturation, Multistep analysis

## Abstract

By affecting the ovarian pool of follicles and their enclosed oocytes, heat stress has an impact on dairy cow fertility. This study aimed to determine how heat shock (HS) during in vitro maturation affected the ability of the bovine cumulus-oocyte complexes (COCs) to develop, as well as their metabolism of amino acids (AAs). In this study, COCs were in vitro matured for 23 h at 38.5 °C (control; n = 322), 39.5 °C (mild HS (MHS); n = 290), or 40.5 °C (severe HS (SHS); n = 245). In comparison to the control group, the MHS and SHS groups significantly decreased the percentage of metaphase-II oocytes, as well as cumulus cell expansion and viability. The SHS decreased the rates of cleavage and blastocyst formation in comparison to the control and MHS. Compared to the control and MHS-COCs, the SHS-COCs produced significantly more phenylalanine, threonine, valine, arginine, alanine, glutamic acid, and citrulline while depleting less leucine, glutamine, and serine. Data showed that SHS-COCs had the highest appearance and turnover of all AAs and essential AAs. Heat shock was positively correlated with the appearance of glutamic acid, glutamine, isoleucine, alanine, serine, valine, phenylalanine, and asparagine. Network analysis identified the relationship between HS and alanine or glutamic acid, as well as the relationship between blastocyst and cleavage rates and ornithine. The findings imply that SHS may have an impact on the quality and metabolism of AAs in COCs. Moreover, the use of a multistep analysis could simply identify the AAs most closely linked to HS and the developmental competence of bovine COCs.

## Introduction

The highest ambient temperature that high milk-yielding dairy cows can tolerate while still maintaining a constant body temperature (38.5 °C) is between 25 and 26 °C (Berman et al. 1985). Dairy cows are highly sensitive to high temperatures (Bernabucci et al. 2014). The body temperature of lactating cows rises to almost 40 °C in environments with high temperatures (Sartori et al. 2002). Heat stress remains a costly problem for the dairy industry, despite improvements in cooling systems and environmental control during the hotter seasons (St-Pierre et al. 2003).

Heat stress can reduce fertility in lactating dairy cows (Badinga et al. 1985; Wolfenson et al. 1995) due to its detrimental effects on follicular growth, secretion of hormones (Badinga et al. 1985), endometrial functionality (Malayer et al. 1988), uterine blood flow (Roman-Ponce et al. 1978), oocyte competence (Rocha et al. 1998; Al-Katanani et al. 2002), and the development of pre-implantation embryos (Putney et al. 1989; Ealy et al. 1993). Oocyte maturation is hampered, and cellular and molecular components crucial for embryo development are damaged, as part of the mechanism by which heat shock (HS) reduces oocyte quality (Gharibzadeh et al. 2014). Also, HS has been shown to decrease the percentage of bovine oocytes that progress to metaphase II (MII) (Payton et al. 2004; Roth and Hansen 2005; Roth 2015). Bovine oocyte maturation is accelerated by HS, and cortical granule migration is faster in heat-shocked oocytes (41.0 °C) (Edwards et al. 2005). It has been demonstrated that HS causes oxidative stress in bovine oocytes, raising reactive oxygen species levels, causing DNA damage, and inducing apoptosis or dysfunction of cellular organelles like mitochondria (Edwards and Hansen 1996; Edwards et al. 2005, 2009; Roth and Hansen 2005; Roth 2015). These all have the potential to reduce the quality and viability of bovine oocytes during in vitro maturation (IVM).

Exposing bovine oocytes to severe HS (SHS, 41–42 °C) revealed changes in gap junction communications between oocytes and cumulus cells (Campen et al. 2018). Fully developed oocytes secrete paracrine substances that enhance cumulus cells to uptake amino acids (AAs) that the oocytes themselves are not very good at transporting. Gap junctions are probably used to deliver these AAs to the oocytes (Eppig et al. 2005).

It has been demonstrated that the synthesis and use of AAs during IVM affect the quality of the bovine oocytes (Hemmings et al. 2012; Kowsar et al. 2018, 2020). In bovine oocytes with less developmental competence, Hemmings et al. (2012) found that there was a greater turnover of AAs. They found that glutamine (Gln), arginine (Arg), and asparagine (Asn) were the most depleted AAs, whereas oocytes with lesser developmental competence released alanine (Ala) and glycine (Gly) into the medium at a greater rate (Hemmings et al. 2012). A greater appearance/depletion of total AAs was observed in oocytes that could not cleave compared to oocytes that could (Hemmings et al. 2012). We previously observed a more pronounced depletion of AAs and a lesser rate of competence when bovine cumulus-oocyte complexes (COCs) matured in vitro under urea stress, a dietary stressor (Kowsar et al. 2018).

Growing attention is being paid to the metabolic profile of media used for human and bovine embryo culture and oocyte maturation ( Hemmings et al. 2012, 2013). The metabolism of AAs in HS-treated bovine COCs, however, is not well understood. This study aimed to examine the effects of HS during IVM on the depletion/appearance of AAs, as well as the subsequent developmental competence of the bovine COCs. Furthermore, using a multistep methodology, we sought to identify the AAs that were most closely related to the developmental competence of bovine COCs and HS.

## Materials and methods

All chemicals and media were prepared from Sigma Aldrich Chemical Co. (St. Louis, MO), unless otherwise stated.

### Research ethics

Animal experiments were conducted in accordance with the Guiding Principles for the Care and Use of Research Animals developed by the Isfahan University of Technology, Iran. The protocol and methods were approved by the Committee on the Ethics of Animal Experiments of the Isfahan University of Technology (No. 390132). The datasets are available from the corresponding author upon reasonable request.

### Recovery of COCs and IVM

From the slaughterhouse to the laboratory, bovine ovaries were transported in a Thermo box containing 0.9% NaCl, 0.1% penicillin, and streptomycin at a temperature of approximately 30 °C. According to previous works (Assidi et al. 2008; Green et al. 2016), the ovaries were rinsed in sterile 0.9% NaCl containing 0.1% penicillin and streptomycin at 30 °C, and then placed into sterile saline until aspiration using an 18 G needle connected to a vacuum pump (80 mm Hg). Ovaries were then punctured, and using a stereomicroscope (Olympus, Tokyo, Japan), only COCs with more than three layers of compact cumulus cells were chosen. Next, COCs were washed in N-(2-hydroxyethyl) piperazine-Nʹ-2-ethanesulfonic acid (HEPES)-buffered tissue culture medium 199 (TCM-199) (Thermo Fisher Scientific, Grand Island, NY, USA) supplemented with 50 mg/mL kanamycin and 50 mg/mL heparin. Following two rounds of washing in HEPES-TCM-199, COCs (n = 857) were divided into one of 3 groups and incubated in a maturation medium (M199). Experimental groups were (1) the control group (38.5 °C, n = 322 COCs; repeated over 8 independent days with 4 replicates of 10 COCs each); (2) the mild HS group (MHS, 39.5 °C, n = 290 COCs; repeated over 7 independent days, with 4 replicates of 10–12 COCs each); and (3) the severe HS group (SHS, 40.5 °C, n = 245 COCs, repeated over 6 independent days with 4 replicates of 10–11 COCs each). Then, COCs were allowed to mature for 23 h (Uhde et al. 2018; Aardema et al. 2013) in 50-µL droplets of maturation medium containing TCM-199 (Thermo Fisher Scientific, Grand Island, NY, USA) supplemented with sodium pyruvate (2.5 mM), L-Gln (1 mM), penicillin (100 IU/mL), streptomycin (100 µg/mL), fetal bovine serum (FBS, 10% v/v, Atlanta Biologicals, Lawrenceville, GA), epidermal growth factor (EGF; 100 ng), follicle-stimulating hormone (FSH; 10 µg/mL, Follitropin; Bioniche Animal Health, Belleville, ON, Canada), luteinizing hormone (LH;10 µg/mL), estradiol-17β (1 µg/mL), and cysteamine (0.1 mM) in 35 mm cell culture dishes (Falcon brand, BD Biosciences, San Jose, CA, USA) at 38.5 °C under 5.0% CO_2_, 20% O_2_ with balanced N_2_ and maximum humidity under mineral oil. Following the incubation period, the oocytes were recovered, the maturation medium was collected and kept at − 80 °C until AA analysis, and the viability of cumulus cells, as well as nuclear maturation, was evaluated.

### Assessment of oocyte nuclear maturation

**Fig. 1:**
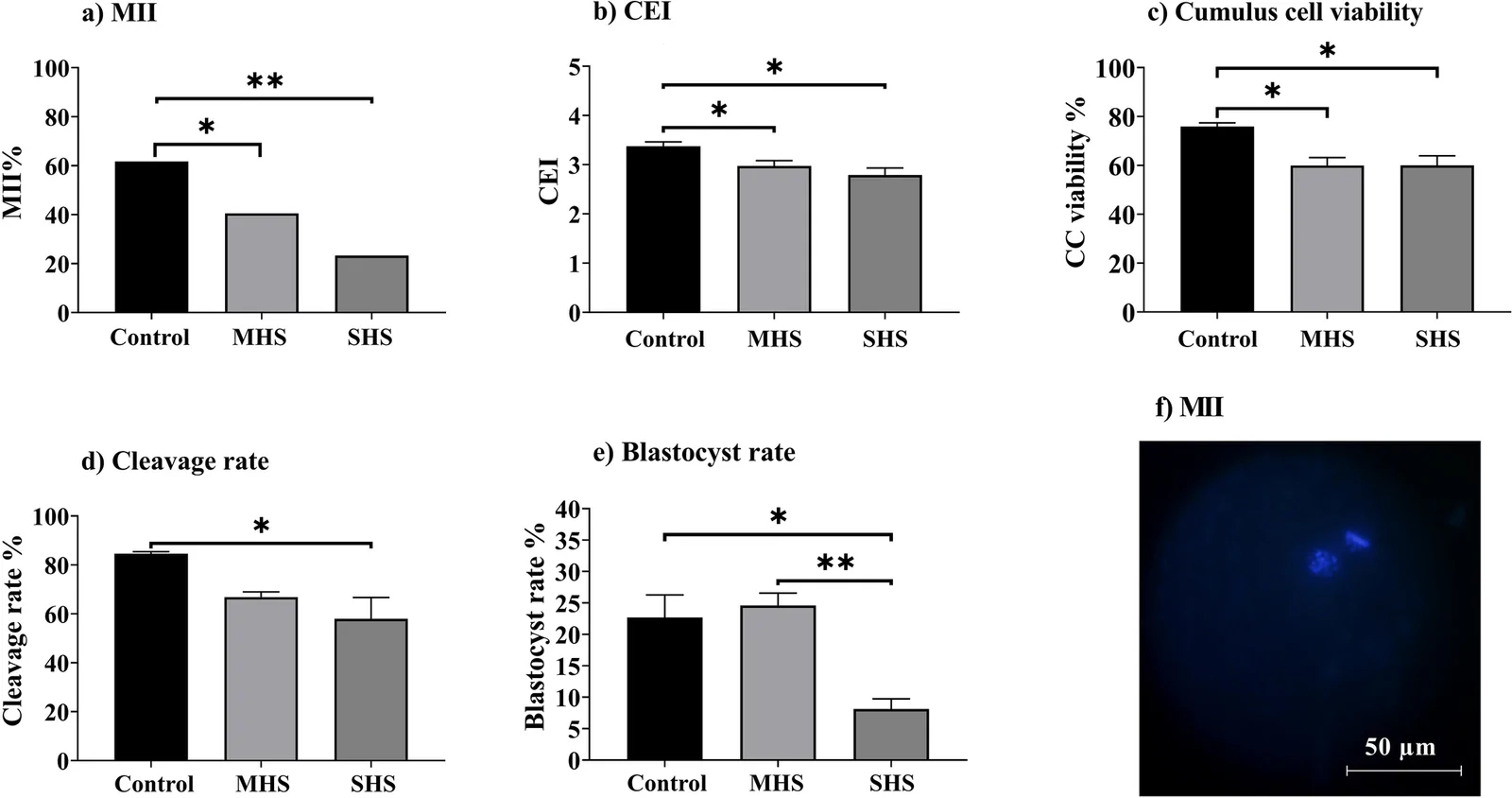
Effect of heat shock on **a** nuclear maturation of bovine oocytes; **b** cumulus expansion index; **c** the viability of cumulus cells; **d** cleavage rate of the resultant zygotes (3 days after in vitro fertilization); and **e** blastocyst rate of resultant embryos (8 days after IVF). **f** Representative image of a bovine oocyte at MII stage with chromosomes and extrusion of first polar body. Bovine COCs matured for 23 h at 38.5 (control), 39.5 (mild heat shock, MHS), or 40.5 °C (severe heat shock, SHS). MII: metaphase II; CEI: cumulus expansion index. Mean + SEM. Asterisks indicate significant differences between groups *P < 0.05; **P < 0.01

According to the previous studies (De Los Reyes et al. 2005; Rovani et al. 2023), COCs were harvested after 23 h of IVM, 300 IU/mL hyaluronidase was added, and cumulus cells were then removed by mechanical stirring. The denuded oocytes were fixed for 20 min at room temperature in 4% paraformaldehyde before being moved to a 0.5% Triton X-100 in phosphate-buffered saline (PBS) solution. Denuded oocytes were incubated with 10 µg/mL of bisbenzimide (Hoechst 33,342) for 15 min in order to stain DNA. Under UV light (wavelength of 340–380 nm) in a fluorescence microscope, oocytes with chromatin configuration consistent with the MII stage were considered mature. A total of 81, 57, and 60 denuded oocytes were used to measure the nuclear maturation in the control, MHS, and SHS groups, respectively, over 4 independent days, with 2 replicates of 7–10 denuded oocytes each. Estimates were made regarding the proportion of oocytes that reached the MII stage (Fig. 1).

### Assessment of the cumulus cell expansion

**Fig. 2:**
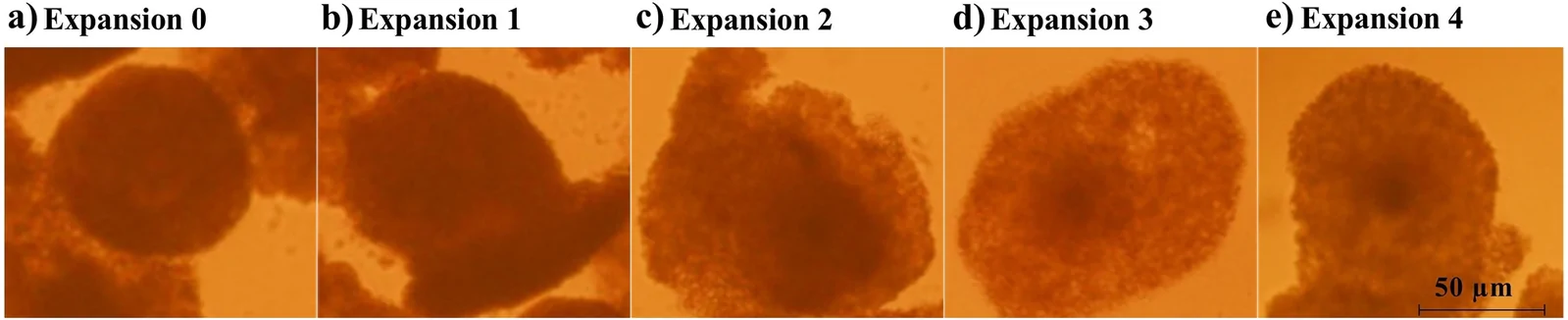
Representative images of the cumulus expansion in the bovine cumulus-oocyte complexes using a 5-point scale as follows: score 0 shows no expansion (**a**); score 1 shows the minimal expansion observable (**b**); score 2 shows expansion of the outer cumulus cell layers (**c**); score 3 shows expansion of all cumulus cell layers except the corona radiate (**d**); and score 4 shows complete expansion of cumulus cell layers (**e**)

To examine the morphology of cumulus expansion both before and after maturation, oocytes were put under an inverted microscope (Nikon Eclipse Ti). Then, using Image J software (Version 1.46; National Institutes of Health, Bethesda, MD, USA), the cumulus area from 60 COCs in each treatment group was calculated and normalized to the control group (Ferronato et al. 2023). To assess the cumulus expansion, 180 COCs (60 COCs per treatment) were used over the course of 3 separate days, with 2 replicates of 10 COCs each. As determined by earlier publications, the cumulus expansion index (CEI) was calculated (Fagbohun and Downs 1990; Vanderhyden et al. 1990). By assigning a score on a 5-point Likert scale, the CEI was assessed for each treatment (Downs 1989). Accordingly, COCs with a score of 0 had either completely lost or only some of their cumulus cells. Also, COCs, that had no expansion but had a spherical cumulus cell, were given a score of 1. Score 2 was indicated by the expansion that had taken place in the cumulus cells’ outer surface layers. Except for the corona radiata, all layers of COCs with a score of 3 had distinctly expanded. The corona radiata and the rate of expansion were both highest at score 4 (Fig. 2).

### Assessment of the cumulus cells’ viability

The viability of the cumulus cells was examined by incubating COCs (n = 245) under three different conditions: control (n = 82 COCs), MHS (n = 84 COCs), and SHS (n = 79 COCs) for 23 h. This experiment was repeated over the course of 3 separate days, with 3 replicates of 9–10 COCs each. Cumulus cells were removed from COCs by vortexing for 3 min using a vortex mixer (Reax Top, Heidolph Instruments, Schwabach, Germany) and hyaluronidase (300 IU/mL in TCM-199). The purified cumulus cells were then centrifuged at 100 × g for 5 min. After removing the supernatant, the pellet was resuspended in 1 mL of sterile PBS (BR0014G, Oxoid Limited, Basingstoke, UK). Trypan blue staining was used to stain cells. The resulting mixture was applied to a hemocytometer and left there for 3 min at room temperature. The number of live and dead cells was determined using an inverted microscope (Olympus, Japan).

### In vitro fertilization (IVF) and in vitro culture (IVC)

The IVF and IVC procedures were carried out according to the previous studies (Hosseini et al. 2016; Sharma et al. 2018; Ferronato et al. 2023). In brief, the 23-h matured oocytes (n = 450) were divided into 3 groups (based on the initial experimental classification, without receiving any treatments) and fertilized in 35 mm cell culture dishes. Matured COCs (10 COCs/droplet) were fertilized with sperm in IVF medium (50 μL-droplets) supplemented with AAs. Frozen semen straws (from a single sire, Spermex, GmbH) were thawed at 37 °C for 40 s. Then, the contents were layered onto a gradient (45:90%) of Pure Sperm (Nidacon, Gothenburg, Sweden) and centrifuged at 700 × g for 15 min at room temperature to obtain highly motile spermatozoa. Sperm cells at a concentration of 1 × 10^6^/mL were co-incubated with COCs in the IVF medium for 18 h at 38.5 °C, 5% CO2, and in a humidified atmosphere. Next, the oocytes (presumed zygotes) were removed from the fertilization drops and serially washed 8–10 times in modified synthetic oviductal fluid (mSOF) media. Presumptive zygotes were collected after 18 h, and cumulus cells were manually removed by vortexing. The resulting embryos were then transferred into cell culture dishes and cultured based on the initial experimental classification (without receiving any treatments) in the culture medium (20-µL droplets) containing mSOF medium. The mSOF was modified before administration to have 1.0 mM alanyl-glutamine, 5.3 mM sodium lactate, 0.5 mM tri-sodium citrate, 2.77 mM myo-inositol, 0.5 mM fructose, 20 μL/mL essential AAs (EAAs, Eagle’s basal medium), and 10 μL/mL nonessential AAs (non-EAAs, minimum essential medium Eagle). Mineral oil was used to cover the culture drops, which were then left in a humidified environment of 90% (vol/vol) N_2_, 5% CO_2_, and 5% O_2_ at a temperature of 38.5 °C for 8 days. The proportion of zygotes that cleaved/developed into the morula (day 3 post-insemination, 8–16-cell stage) and blastocyst (day 7 post-insemination) stages was assessed using a stereomicroscope (Olympus, Tokyo, Japan). Cleavage and blastocyst rates were determined per the initial number of COCs cultured for IVM. This experiment (IVF and IVC) was repeated for 5 independent days, with 3 replicates of 10 presumptive zygotes each (150 matured oocytes or presumptive zygotes per treatment).

### Differential staining of blastocysts

As previously mentioned (Hosseini et al. 2007), hatched blastocysts (on day 8 post-insemination) were stained to ascertain the total cell number (TCN) and individual cell numbers [the inner cell mass (ICM) and trophectoderm (TE)]. Briefly, hatched blastocysts were washed in HEPES-TCM-199 + 5 mg/mL BSA before being exposed to 0.5% Triton X-100 and 30 µg/mL propidium iodide in the medium for 1 min. Embryos were then placed in a low-temperature (4 °C) counter-staining solution for 15 min using Hoechst 33,342 (10 µg/mL) diluted in ethanol. Then, hatched blastocysts were mounted, and they were examined under a fluorescent microscope (Olympus, Japan). A total of 36 hatched blastocysts (12 hatched blastocysts per treatment) were examined over the course of 3 separate days, with 4 hatched blastocysts each in order to assess the differential cell number.

### Amino acids analysis

Maturation medium droplets were taken separately to measure the concentration of AAs after a 23-h incubation period. The experiment was repeated 6 times, using 2–3 different media for each treatment at each time point (a total of 17–18 different media). Based on our previous research (Kowsar et al. 2018, 2020), the HPLC method (HyperClone ODS C 18, 250 mm, 4.6 mm, 5 m, Agilent 1100, Agilent Technologies, Waldbronn, Germany) was used to determine AAs in the 23-h spent media and fresh maturation media (n = 3). For HPLC analysis, a flow rate of 1.2 mL/minute was used with fluorescence detection after pre-column derivatization with ortho-phthalaldehyde (OPA). The fluorescent signal was 450 nm when it was excited at 348 nm. The same procedure for analysis was used on all blanks and test medium, which were immediately stored at − 80 °C. As a control sample (blank), COC-free maturation media were used. Indeed, maturation medium droplets devoid of oocytes were cultured in parallel with the test samples at three different temperatures (38.5, 39.5, and 40.5 °C) for 23 h. The concentration of AAs did not significantly differ between the blanks and remained constant throughout the experiment (data not shown).

The peak regions of the samples and the reference AA combination were compared to determine AA concentrations. All peak signals were normalized using peak signals from internal standards. As internal standards, 25 mM sarcosine (Hewlett-Packard GmbH, FRG, Switzerland) and 25 mM norvaline (Hewlett-Packard GmbH, FRG, Switzerland), both in 0.1 M HCl solution, were used. Ortho-phthalaldehyde has been shown to have a poor reaction to cystine and none to proline when the thiol component is present; the OPA technique was used in this study (Walker and Mills 1995). Due to the conversion of Asn to aspartic acid (Asp) during sample processing, the assay for Asn was unlikely (D’Mello 2009). Phenylalanine (Phe), isoleucine (Ile), leucine (Leu), valine (Val), methionine (Met), threonine (Thr), lysine (Lys), Arg, tyrosine (Tyr), histidine (His), and tryptophan (Trp) were the EAAs in this study. Semi-EAAs were serine (Ser), Gly, and Gln. Non-EAAs were Asp, Asn, Ala, citrulline (Cit), ornithine (Orn), and glutamic acid (Glu), according to previous works (D’Mello 2009; Hou et al. 2015; Kowsar et al. 2018, 2020). In this study, we considered some AAs, such as Arg and Tyr, as EAAs while they are classified as semi-EAAs for cows. Of note, some cell types, like cumulus cells or oocytes, cannot produce these AAs. As a result, these AAs are regarded in this situation as EAAs (Wu et al. 2009).

### Calculation of AA depletion/appearance

According to previous studies, the depletion/appearance of AAs was calculated at picomoles per oocyte per hour ( Houghton et al. 2002; Hemmings et al. 2012, 2013; Kowsar et al. 2018, 2020). The differences in the concentration of AAs between the fresh medium (at 0 h of incubation) and the spent-maturation medium (after 23-h incubation) were used to calculate the depletion or appearance of each AA. Negative values after a 23-h incubation period indicate “depletion”, which is the disappearance of AAs from the maturation medium. Positive values represented the increased AA levels in the maturation medium, referred to as “appearance”. Total depletion and total appearance were calculated by summing the negative values (depletion) and positive values (appearance) obtained for AAs. To calculate the “total turnover” of all AAs, the “total depletion” and “total appearance” were summed. Subtracting “total depletion” from “total appearance” yielded an estimate of the “total net balance” of all AAs.

### Accuracy of AA analysis

To evaluate the efficacy of the AA analysis, we calculated the mean recovery of one biological medium (i.e., blood plasma) containing a specific amount of AAs. To this, each amino acid standard was mixed separately to a final concentration of 60.0 M in a pooled plasma sample, and the recovery of the amino acids was calculated as the difference between the spiked and unspiked plasma samples (Frank and Powers 2007). So, accuracy was defined as the agreement between measured and actual values of AAs in a reference medium. For all AAs that were measured, the average recovery rate was 102.6%. The precision ranged from 93.1 to 106.9%, with Asp being the exception (112.4%). As a result, the range for all AAs was set at 90–115%. To test the precision of the method, we also looked at repeatability by injecting the same sample of fresh maturation medium 4 times in a row. We did this by calculating the relative standard deviation (RSD) for each AA. The RSD values were within an acceptable range of 1.12–3.36% (Jajic et al. 2013).

### Bivariate Kendall’s tau correlation, PCA, and network analysis

The bivariate study employed Kendall’s tau correlation to determine the association between variables. The principal component analysis (PCA) is performed to clarify the concomitant interactions and intricate connections or multi-collinearity between variables. This method has the potential to minimize the number of variables while maintaining the majority of the critical information. Multiple data dimensions were reduced to two dimensions in this study, and the PCA biplot was created with PAST software. Associations in the PCA biplot are presented as directional vectors and defined by the angle between vectors. Vectors with < 45° angles exhibited a positive connection, vectors with perpendicular angles (approaching 90°) had no link, and vectors heading in the reverse direction (approaching 180°) had a negative correlation. The Spearman’s rho and Pearson similarity indices were used in combination with the Fruchterman-Reingold algorithm as a force-directed layout algorithm in network analysis. This method constructs a network based on the frequency with which nodes are linked. In this study, the PAST tool (available at: http://folk.uio.no/ohammer/past) was used to do ANOVA, Kendall’s tau correlation, PCA, and network visualization analyses.

### Statistical analysis

The data were found to be normal by the Anderson–Darling test. Tukey’s multiple comparisons test was used to compare the data on AA depletion and appearance and cumulus cell viability across treatments. A nonparametric approach, the Kruskal–Wallis test, was used to statistically analyze the cumulus expansion index. The chi-squared test was used to statistically analyze categorical variables, such as oocytes at the MII stage. All data were shown as mean ± standard error of the mean (SEM). The differences between groups were considered significant at *P* < 0.05. The PAST tool and GraphPad Prism software (version 8.0) were used to analyze the data.

## Results

### Heat shock reduces oocyte maturation rate and competence

Compared to the control group, the SHS group had a significantly greater percentage of COCs that did not reach the MII stage (61.73% vs. 23.33%, *P* < 0.001, Fig. 1a). The lesser proportion of MHS-COCs reached the MII stage (40.54%) compared to the control COCs (*P* = 0.03, Fig. 1a). Figure 1f shows a representative image of a COC in the MII phase. As seen in Fig. 1b, the CEI values decreased in the SHS and MHS groups compared to the control group (2.92 ± 0.1, 2.95 ± 0.1, and 3.40 ± 0.1; respectively, *P* < 0.01). Figure 2 shows the representative images of the expansion of cumulus cells. The MHS (60.0%) and SHS (60.0%) groups had significantly lesser cumulus cell viability rates than the control group (75.9%) (*P* = 0.02; Fig. 1c).

**Fig. 3:**
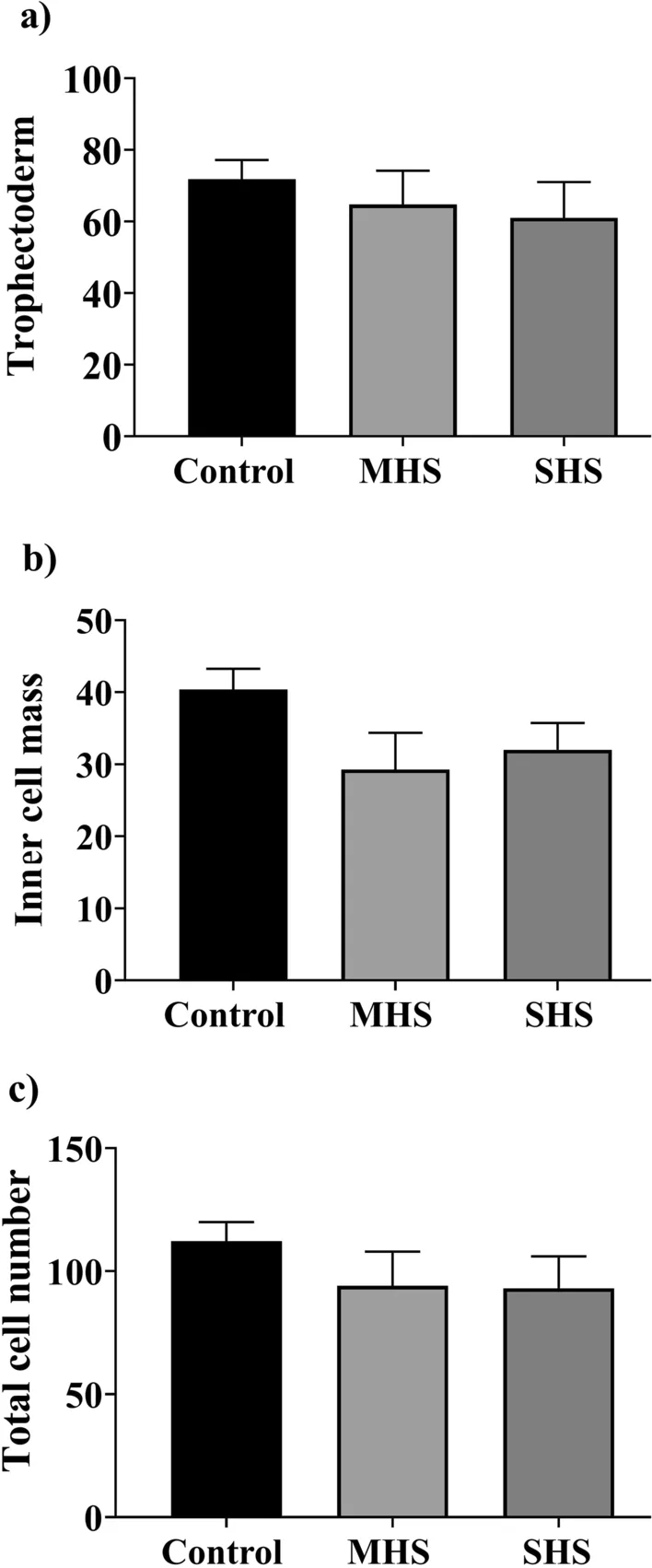
Effect of heat shock on (**a**) trophectoderm cell number; (**b**) inner cell mass; and (**c)** the total cell number of blastocysts resulted from bovine cumulus-oocyte complexes matured for 23 h at 38.5 (control), 39.5 (mild heat shock, MHS), or 40.5 °C (severe heat shock, SHS). Mean + SEM

According to the findings, SHS (58.0%) decreased (*P* < 0.05) the cleavage rate compared to the MHS (66.9%) and control groups (84.6%; Fig. 1d). Additionally, COCs in the SHS group (8.1%) had a lesser rate of blastocyst formation when compared to the control (22.7%) and MHS (24.6%) groups (*P* < 0.01, Fig. 1e). Figure 3 showed that the ICM, TE, and TCN between groups did not differ significantly.

### Heat shock affects the depletion/appearance of AAs by COCs during IVM

**Fig. 4:**
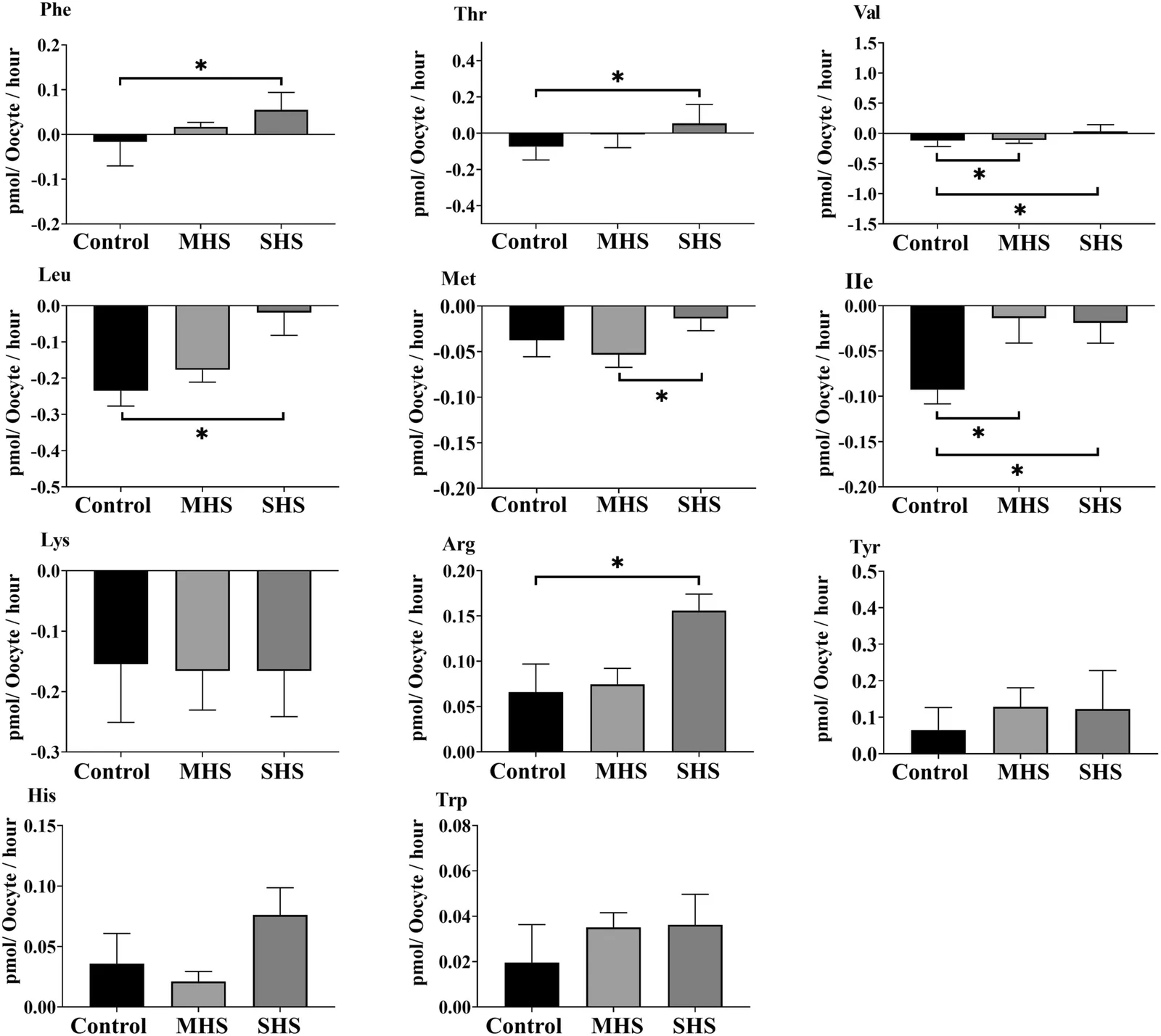
Effect of heat shock on the net depletion/appearance of essential amino acids by bovine cumulus-oocyte complexes after 23-h maturation at 38.5 (control), 39.5 (mild heat shock, MHS), or 40.5 °C (severe heat shock, SHS). Mean + SEM. Significant differences between groups are indicated by asterisks. *indicates *P* < 0.05

**Fig. 5:**
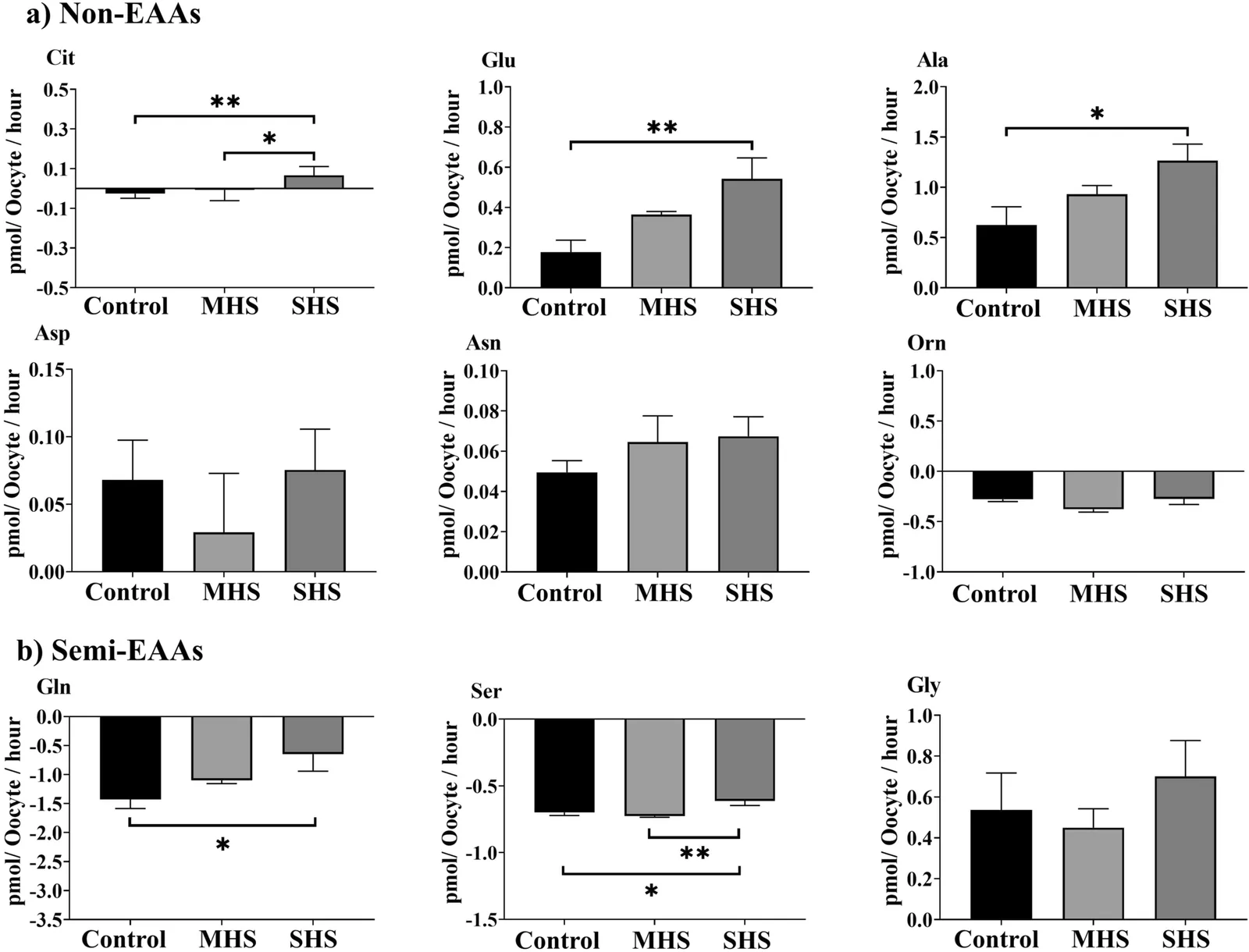
Effect of heat shock on net depletion/appearance of (**a**) non-essential amino acids (EAAs) and (**b**) semi-EAAs by bovine cumulus-oocyte complexes after 23-h maturation at 38.5 (control), 39.5 (mild heat shock, MHS), or 40.5 °C (severe heat shock, SHS). Mean + SEM. Significant differences between groups are indicated by asterisks. *indicates *P* < 0.05; **indicates *P* < 0.01

Compared to the control- or MHS-COCs, the SHS-COCs released significantly more EAAs, including Phe (*P* < 0.01), Thr (*P* = 0.04), Val (*P* < 0.01), and Arg (*P* = 0.03), into the culture medium and depleted less Leu (*P* < 0.01) and Ile (*P* = 0.04) from the medium (Fig. 4). The SHS group produced more non-EAAs, including Ala (*P* = 0.03), Glu (*P* < 0.01), and Cit (*P* < 0.01), compared to the control or MHS groups (Fig. 5a). The SHS group depleted less Gln (*P* = 0.03) and Ser (*P* < 0.01) than the control or MHS groups did (Fig. 5b).

### Depletion/appearance of all AAs

**Fig. 6:**
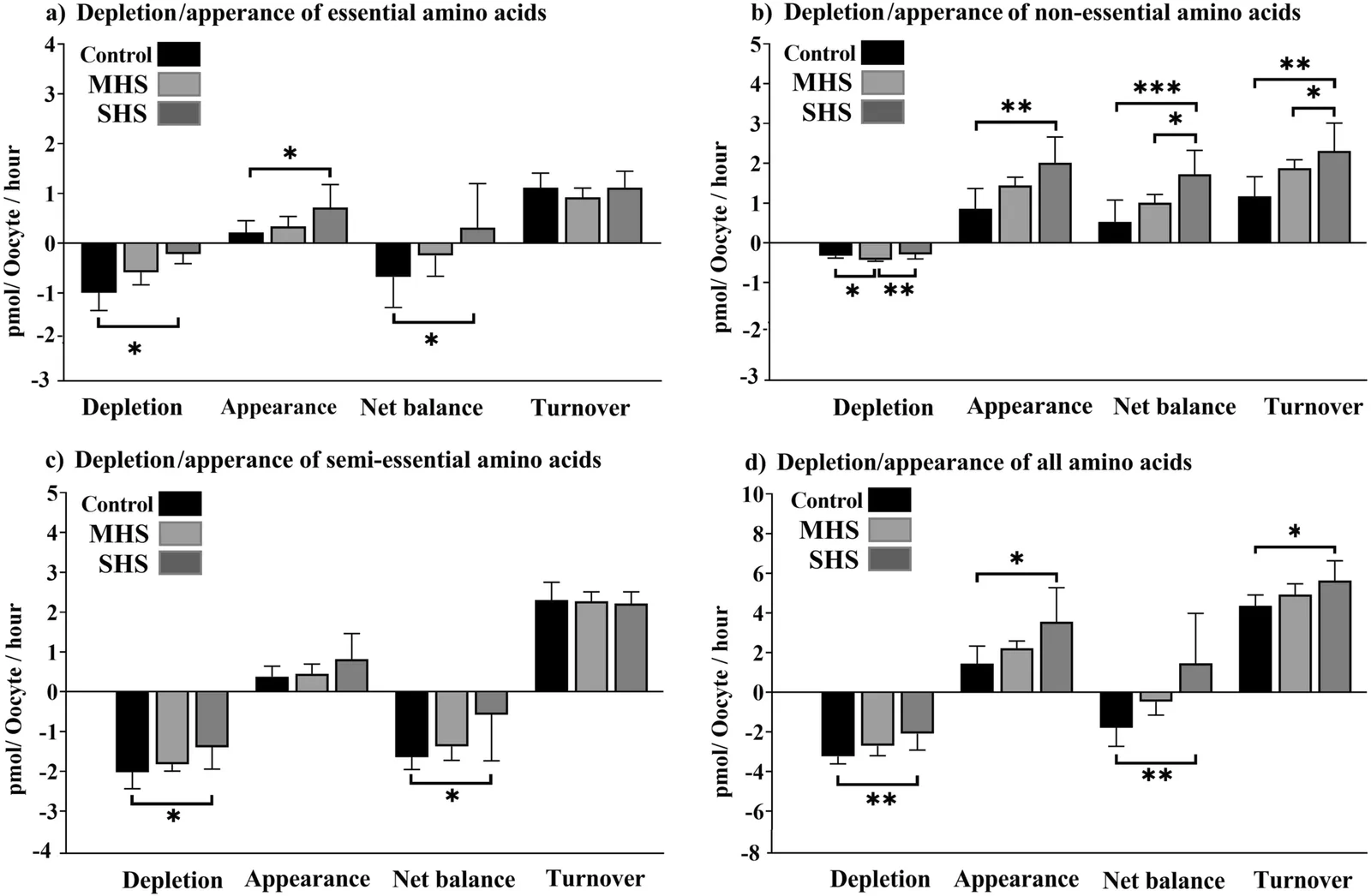
Effect of heat shock on the total depletion, appearance, net balance, and turnover of essential amino acids (**a**); non-essential amino acids (**b**); semi-essential amino acids (**c**); and all amino acids (**d**) by bovine cumulus-oocyte complexes after 23-h maturation at 38.5 (control), 39.5 (mild heat shock, MHS), or 40.5 °C (severe heat shock, SHS). Mean + SEM. Asterisks indicate significant differences between groups: *indicates *P* < 0.05; **indicates *P* < 0.01; ***indicates *P* < 0.001

The SHS group had lesser levels of EAA depletion than the control or MHS groups (*P* = 0.04). In comparison to the control or MHS groups, the SHS group released more EAAs (*P* = 0.03) into the media. Compared to the control or MHS groups, the net balance of AAs was also noticeably greater in the SHS group (*P* = 0.04, Fig. 6a). More non-EAAs were depleted in the MHS group compared to the control and SHS groups (*P* = 0.006) (Fig. 6b). In comparison to the control and MHS groups, the SHS group had a greater appearance (*P* < 0.001), net balance (*P* < 0.001), and turnover (*P* < 0.01) of non-EAAs (Fig. 6b). The SHS-COCs had a lesser depletion (*P* = 0.03) and net balance (*P* = 0.03) of semi-EAAS compared to the control-COCs (Fig. 6c). The total AA depletion was significantly lesser in the SHS group compared to the control or MHS groups (*P* < 0.01, Fig. 6d). The SHS group outperformed the control and MHS groups in terms of appearance (*P* = 0.01), net balance (*P* < 0.01), and turnover of all AAs (*P* = 0.02, Fig. 6d).

### The association of AA metabolism with HS and COC’s developmental competence

**Fig. 7:**
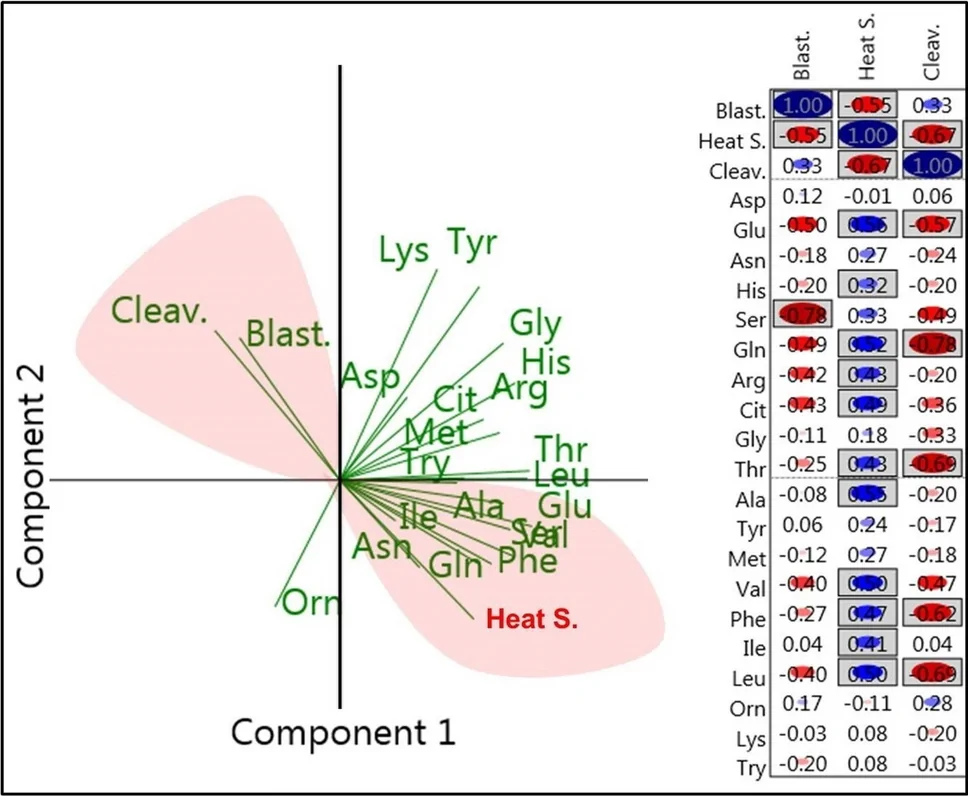
**a** Biplot of PCA derived from the amino acid metabolism and developmental competence of the bovine cumulus-oocyte complexes. Vectors with close angles (< 45°) indicate a strong correlation, vectors that are perpendicular indicate no correlation, and vectors in opposite directions (approaching 180°) indicate a negative correlation. **b** The bivariate study employed Kendall’s tau correlation to determine the association between amino acid metabolism, heat shock, and developmental competence of COCs. Red and blue circles indicate negative and positive associations, respectively. Boxed rectangles indicate the statistical significance at *P* < 0.05. *Blast* blastocyst rate, *S* shock, *Cleav* cleavage rate

According to the PCA analysis, HS was closely positively associated with the metabolism of the AAs, including Ala, Glu, Ser, Val, Ile, Phe, Gln, and Asn (Fig. 7a, right, lower red field), while the rates of cleavage and blastocyst formation were negatively associated with the metabolism of these AAs (Fig. 7a, left, the upper red field). The metabolism of the AAs, including Glu, His, Gln, Arg, Cit, Thr, Ala, Val, Phe, Leu, and Ile, by COCs in the maturation medium was found to be positively correlated with HS, according to Kendall's tau analysis (*P* < 0.05, Fig. 7b). The metabolism of Ser by COCs was inversely correlated with consequent blastocyst formation (*P* < 0.05, Fig. 7b). The metabolism of Glu, Gln, Thr, Phe, and Leu by COCs in the maturation medium was negatively correlated with the cleavage rate (*P* < 0.05, Fig. 7b).

**Fig. 8:**
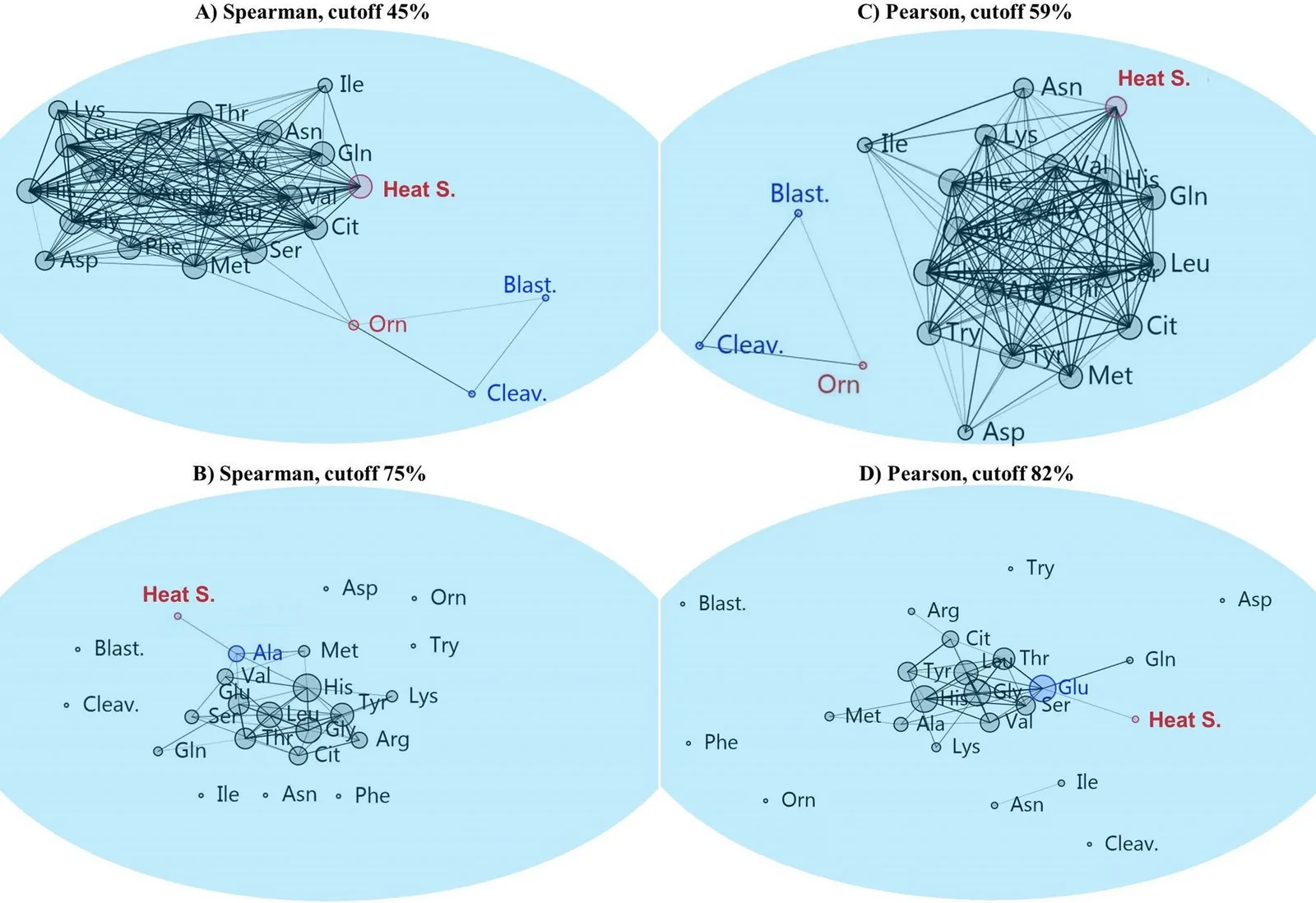
The Spearman’s rho (**a, c**) and Pearson (**b, d**) similarity indices were used in combination with the Fruchterman–Reingold algorithm as a force-directed layout algorithm in the network analysis. This method constructs a network based on the frequency with which nodes are linked. The panels show the most closely related amino acid/s metabolism in cumulus-oocyte complexes during in vitro maturation to (**a**, **b**) blastocyst rate and cleavage rate or (**c**, **d**) heat shock. *Blast* blastocyst rate, *Cleav* cleavage rate

The network analysis using either Spearman's rho or Pearson similarity revealed that, at the highest cut-off points, the cleavage and blastocyst formation rates were related to Orn (Fig. 8a, b). Using Spearman’s rho-based network analysis or Pearson similarity-based network analysis, at the highest cut-off points, HS was linked to Glu or Ala, respectively (Fig. 8c, d).

## Discussion

This study demonstrated that HS had an impact on the quality, developmental competence and the depletion/appearance of AAs in bovine COCs in vitro. Moreover, using a multistep approach, we observed that the metabolism of Orn by COCs was closely related to COCs’ developmental competence and that the metabolism of Ala and Glu by COCs was closely related to HS.

It was expected that fewer COCs receiving SHS treatment (40.5 °C) would progress to the MII stage, as this result was in line with earlier studies (Nabenishi et al. 2012; Maya-Soriano et al. 2013). An earlier study found that even a short exposure to HS can disrupt the genomic control of oocyte maturation (Kowalczyk et al. 2021). It has been observed that in mice, HS impairs spindle formation during metaphase I and hinders progression to MII (Baumgartner et al. 1987). The current findings suggested that SHS lessened the ability of prophase I-arrested oocytes to escape into the MII stage.

In addition, SHS-COCs showed lesser cumulus cell viability, cumulus expansion index, and developmental competence compared to the control group. The results were completely in line with earlier studies that used 40.0 °C (Roth and Hansen 2005; Edwards et al. 2009) or 41 °C (Roth and Hansen 2005) during the 24-h IVM in the bovine COCs (Roth and Hansen 2005; Edwards et al. 2009). As opposed to mild exposure, it has been demonstrated that exposure to SHS increases cell susceptibility to apoptosis (Pöhland et al. 2020). Bovine oocytes have been demonstrated to undergo apoptosis in response to HS (Khan et al. 2020). This may help to partially explain why SHS led to lesser cumulus cell viability in this study.

As previously mentioned, SHS decreased the CEI and the percentage of oocytes that reached the MII stage. It has been shown that some substances, such as cumulus-expansion enabling factors (CEEFs), are secreted by fully grown oocytes and are essential for expansion to occur (Vanderhyden et al. 1990; Ochsner et al. 2003; Su et al. 2003). These findings implied that SHS might cause insufficient oocyte growth as cumulus expansion decreased (Chen et al. 1993). Therefore, the current research confirmed that SHS impaired oocyte maturation (i.e., MII) and cumulus cells’ expansion and viability; all of which could affect the quality and developmental competence of bovine COCs.

The present findings showed that compared to the control group, bovine COCs that matured at SHS had a significantly greater appearance, net balance, and turnover of all AAs and EAAs. High levels of AA turnover have been linked to lesser quality of bovine oocytes and embryos (Leese et al. 2008; Hemmings et al. 2012). Recently, we found that urea-reduced oocyte competence was linked to increased AA turnover and depletion (Kowsar et al. 2020). Therefore, the findings suggested that SHS-reduced oocyte competence might be related to increased total appearance, net balance, and total turnover of all AAs in COCs.

The most closely related AAs to HS and COCs competence were sought after using a combination of ANOVA, Kendall’s tau correlation, PCA, and network analyses in this study. First, an ANOVA analysis revealed that SHS-COCs released more Phe, Val, Ala, and Glu while depleting less Leu, Ile, Ser, and Gln. According to the findings of Kendall’s tau analysis, these AAs had a positive correlation with HS despite having a negative correlation with blastocyst formation and cleavage rates. The results of Kendall's tau analysis were supported by PCA analysis, which can be used to determine the interrelationships between AAs. The PCA revealed a strong and positive association between HS and the metabolism of these AAs in COCs. Additionally, PCA analysis showed a strong negative association between the metabolism of these AAs and COCs’ developmental competence. At the final stage of analysis, using network analysis at the highest cut-off point of 75% based on Spearman’s Rho (a nonparametric measure of association), the metabolism of the Ala in COCs was found to be associated with the HS. As previously mentioned, the SHS-COCs group with the highest appearance of Ala displayed the lowest rates of blastocyst formation and cleavage. Hemmings et al. (2012) reported that bovine oocytes that did not cleave produced more Ala than oocytes that did, which is consistent with our findings. It has been shown that some AAs, such as Ala, which are poorly transported into oocytes, are taken up by cumulus cells (Su et al. 2003). Alanine should first be taken up by cumulus cells, and then it should be transferred to the oocyte by gap junctions (Su et al. 2003). In porcine cumulus cells and oocytes, as well as bovine oocytes, it has been demonstrated that HS disrupts gap junctions (Hamada et al. 2003; Campen et al. 2018). The results implied that the reduced viability of cumulus cells caused by SHS might result in a lesser amount of Ala entering the oocytes to perform its physiological functions. Nissim et al. (1992) proposed that Ala serves a functional purpose in both protecting cells from stress-related cell damage and promoting gene expression that is responsible for stress protein synthesis. The total amount of intracellular protein produced by maturing oocytes has also been shown to be decreased by HS (Lannett and Hansen, 1996). As a result, oocytes might produce more Ala to make up for this need, which could lead to more Ala being released into the maturation medium; the greater Ala concentrations in the SHS group in the present study might be explained by this.

In the current study, SHS-COCs released greater levels of Glu into the maturation medium than did control or MHS-COCs. The results obtained using Kendall's tau and PCA analyses showed that Glu concentrations were positively correlated with HS and negatively correlated with cleavage and blastocyst formation rate. Miao et al. (2022) demonstrated that the main factor affecting the distinction between high-quality and low-quality embryos is Gln, which can be metabolized by conversion to glutamate. Notably, the Pearson-based network analysis showed that Glu was the most correlated AA with HS at the highest cut-off point of 82%. However, Ala was identified as the most correlated AA by Spearman’s Rho-based network analysis. It should be noted that in complex networks, the rank correlations (such as Spearman’s rho) are thought to perform better than Pearson correlation (Shao et al. 2018). To be able to recognize and validate the best results, we recommend that data be analyzed using a variety of methods.

As previously mentioned, we found that Glu released from SHS-COCs into the maturation medium was greater compared to the control and MHS-COCs groups. In addition, we found that Glu concentrations were negatively correlated with blastocyst and cleavage rates. In contrast, Stokes et al. (2007) found that Glu appearance was a good indicator of developmental competence of COCs. Nucleic acid bases, which can be produced by animal or human cells from some AAs, such as Glu, are shown to be necessary for the completion of meiosis in the maturing oocyte (Salzman et al. 1958). Notably, the increased need for Gln and Glu seen in oocytes that failed to cleave provides purines and pyrimidines for DNA repair (Sturmey et al. 2009). It has been shown that in preimplantation embryos, glutamic acid can function as a signaling molecule, having an impact by activating cell membrane receptors (Špirková et al. 2022). More specifically, glutamic acid can be used as a source of energy, a bridge in transamination reactions, or a starting point for the synthesis of a number of significant molecules, including glutathione, the primary intracellular antioxidant (Brosnan and Brosnan 2013; Špirková et al. 2022). This could imply that HS had an impact on Glu uptake, which is important for a number of cellular processes of oocytes, such as DNA repair, the completion of meiosis and the source of energy. Fewer COCs were able to progress to the MII stage, which raises the possibility that SHS hindered the COCs’ ability to access Glu.

Ornithine was continuously depleted by COCs in all experimental groups, and HS in our study did not affect Orn consumption. Furthermore, Orn metabolism was positively correlated with cleavage and blastocyst formation rates, according to the findings of the network and Kendall’s tau analyses. Previously, we found a positive correlation between the viability of cumulus cells and the turnover of Orn. Further study is required to clarify the relationship between COCs’ consumption of Orn and the rates of cleavage and blastocyst formation.

In the current study, we also found that SHS-COCs with the lowest levels of developmental competence released more Arg than the control and MHS-COCs. Kendall’s tau analysis also demonstrated a positive correlation between HS and Arg appearance, as well as a negative association between Arg appearance and developmental competence of COCs. Nitric oxide production by mammalian cells has been connected to Arg (Cendan et al. 1996). The physiological importance of nitric oxide in oocyte maturation, fertilization, and early embryo development has been established (Taglio and Parma 1999; Shiva et al. 2007; Chen et al. 2008). Also, Goud et al. (2014) showed that nitric oxide was an important component of oocyte quality maintenance in humans. It is possible that SHS-increased Arg appearance, rather than its consumption to produce nitric oxide, might affect in vitro meiotic maturation and oocyte competence (Budani and Tiboni 2021). More research is required, particularly in the areas of nitric oxide and Arg metabolism and bovine oocyte competence under HS conditions.

In our study, SHS-COCs consumed noticeably less Ser compared to the control or MHS-COCs groups. The conversion of Ser to pyruvate during oocyte growth and cytoplasmic maturation has been demonstrated to support gluconeogenesis and increase energy availability (Pelland et al. 2009). By reducing COCs’ Ser consumption, heat shock appeared to have an impact on their ability to access energy, at least in part.

In conclusion, our research showed that SHS had a detrimental effect on the quality and developmental competence of bovine COCs. Additionally, it was found through a variety of statistical analyses that the metabolism of AAs (i.e., Orn, Glu, and Ala) during IVM had a close relationship with either SHS or the developmental competence of bovine COCs.

## Data Availability

The data underlying this article are available in the article.
